# Association Between Air Pollutants and Pediatric Respiratory Outpatient Visits in Zhoushan, China

**DOI:** 10.3389/fpubh.2022.865798

**Published:** 2022-04-04

**Authors:** Wen-Yi Liu, Jing-Ping Yi, Leiyu Shi, Tao-Hsin Tung

**Affiliations:** ^1^Department of Health Policy Management, Bloomberg School of Public Health, Johns Hopkins University, Baltimore, MD, United States; ^2^Institute for Hospital Management, Tsing Hua University, Shenzhen, China; ^3^Shanghai Bluecross Medical Science Institute, Shanghai, China; ^4^Zhoushan Municipal Center for Disease Control and Prevention, Zhoushan, China; ^5^Evidence-Based Medicine Center, Taizhou Hospital of Zhejiang Province Affiliated to Wenzhou Medical University, Linhai, China

**Keywords:** children, air pollutant, respiratory diseases, outpatients, lag pattern

## Abstract

**Objective:**

This study aimed to explore the time-series relationship between air pollutants and the number of children's respiratory outpatient visits in coastal cities.

**Methods:**

We used time series analysis to investigate the association between air pollution levels and pediatric respiratory outpatient visits in Zhoushan city, China. The population was selected from children aged 0–18 who had been in pediatric respiratory clinics for eight consecutive years from 2014 to 2020. After describing the population and weather characteristics, a lag model was used to explore the relationship between outpatient visits and air pollution.

**Results:**

We recorded annual outpatient visits for different respiratory diseases in children. The best synergy lag model found a 10 μg/m^3^ increase in PM_2.5_ for every 4–10% increase in the number of pediatric respiratory outpatient visits (*P* < 0.05). The cumulative effect of an increase in the number of daily pediatric respiratory clinics with a lag of 1–7 days was the best model.

**Conclusions:**

PM_2.5_ is significantly related to the number of respiratory outpatient visits of children, which can aid in formulating policies for health resource allocation and health risk assessment strategies.

## Highlights

- Instruct policies of health allocation and health risk assessment regarding childhood respiratory diseases.
- Help verify the relationship between childhood respiratory problems and air pollutants.
- Indicate for every 10 μg/m^3^ increase in PM_2.5_, the number of childhood respiratory outpatient visits increase by 4–10%.
- Significantly contribute to the existing literature by adding developing country data.
- Fill the research gap of establishing the relationship between air pollutants and respiratory diseases in coastal cities.

## Introduction

In China, the unprecedented economic growth and increased population density have led to the increased severity of environmental problems, especially air pollution (1). Since January 2013, severe haze events have affected 800 million people in 75% of China's cities, restricting sustainable societal and economic growth and threatening public health (2, 3).

Air pollution increases economic loss and respiratory disease morbidity and mortality (4–6). Children are at high risk of environmental stress; additionally, a relationship has been observed between childhood respiratory disease severity and air pollutant concentration (7). Respiratory diseases, such as pneumonia, bronchitis, and asthma, are the most common causes of mortality and morbidity in children worldwide (8). Environmental exposure may lead to low pulmonary function in children, increasing the risk of short-term clinical symptoms and adversely affecting pulmonary function. This subsequently increases the risk of adult chronic respiratory diseases resulting in a significant social burden (9–12).

The relationship between air pollution and children's respiratory health is predominantly established in developed countries. In China, previous studies were mostly conducted in northern cities where air pollution was relatively severe and found a relationship between air pollution exposure and increased outpatient visits in children with respiratory problems (13, 14). Only one study in Shanghai used data from a limited range of air pollutants tested before 2013 (15). Recent studies have found a strong link between air pollution and childhood respiratory problems (16–18).

The Chinese government developed several strategies to address regional air pollution. Since 2013, China has constructed air quality monitoring stations across the country and has gradually released real-time monitoring data of air pollutants such as PM_2.5_ and ozone (O_3_). In 2016, the Central Committee of the Communist Party of China and the State Council released the Health China 2030 Plan, which emphasizes the strengthening of the management of health-related environmental issues (19). This study investigated the relationship between short-term air pollution exposure and daily hospital admissions in children with respiratory diseases to understand the pertinence and refinement of air quality management policies.

## Methods

### Air Pollution and Meteorological Data

Zhoushan City is one of the 27 cities in the central area of the Yangtze River Delta in the northeast of Zhejiang Province. It is a coastal city with a subtropical monsoon climate, a warm winter, cool summer, mild and humid, with sufficient sunlight. By the end of 2018, the annual average PM_2.5_ concentration in Zhoushan was 22 μg/m^3^; additionally, the proportion of days with good daily air quality (AQI) was 94.8%. We collected the average daily records of environmental pollutants (μg/m^3^) from the Zhoushan Environmental Monitoring Center from January 2014 to December 2020, including the average daily levels of PM_2.5_, sulfur dioxide (SO_2_), nitrogen dioxide (NO_2_), carbon monoxide (CO), and O_3_. Additionally, the daily average temperature, relative humidity, and atmospheric pressure of Zhoushan during the same period were obtained from the China Meteorological Data Sharing Service System (http://zshbj.zhoushan.gov.cn/index.html). Finally, the overall air quality data of Zhoushan City was obtained from the comprehensive correction of data collected from Putuo East Port, Dinghai Tain, Feng, and Lincheng New Area.

### Study Population

The population of Zhoushan has stably increased from 1.145 million to 1.176 million between 2014 and 2020 (19). This study selected the Zhoushan Hospital as the research site since it is the only tertiary, first-class general hospital in Zhoushan City and is preferred by residents due to its technology, professionalism, service, and advanced facilities and equipment.

Daily pediatric outpatient records from January 2014 to December 2020 were obtained from the Zhoushan Center for Disease Control and Prevention. Outpatient records for respiratory events were coded using J00–J99 and R04–R09 of the International Classification of Diseases, 10th edition. This study included children aged 0–18 years, regardless of history of hospitalization. All data were anonymized to protect participants' privacy. This observational study was approved by the Institutional Review Board of the Shanghai International Medical Center (SIMC-IRB No: 20210513).

### Statistical Analysis

A generalized additive model with natural splines was constructed to link data by date to explore the association between ambient air pollutants and daily pediatric respiratory outpatients. Additionally, the associations between daily pediatric respiratory outpatients, daily concentrations of ambient air pollutants (PM_2.5_, PM_10_, SO_2_, O_3_, NO_2_, and CO), and meteorological factors (temperature, humidity, and atmospheric pressure) were analyzed. A basic model was constructed without air pollutants. Natural spline functions of time and meteorological parameters were incorporated into the model to adjust for confounding effects, with the day of the week (DOW) as a dummy variable. To prevent model multilinearity, we used Spearman's test to examine the correlation between daily concentrations of ambient air pollutants (20).

The model was examined using Akaike's information criterion (AIC). Additionally, partial autocorrelation functions were used to examine the autocorrelations of the residuals. Synthesizing the results from AIC, PACF, 3 df for the relative humidity, and 6 df for the mean temperature were added in our work as indicator variables. The model is described as follows.


$$\begin{array}{l} \text{log}[E(Y_{t})]=intercept+ns(time,6)+ns(temperature,6)\\ \;+ns(humidity,3)+DOW+\sum_{i=1}^{q}\beta_{i}(X_{i}) \end{array}$$

Where: *E*(*Y*_*t*_) is the number of expected pediatric outpatients at day t; β_*i*_, the association between the log-relative rate of outpatient visits and air pollutant unit increase; *X*_*i*_, the concentration of air pollutants on day t; DOW, the dummy variable; ns (time/temperature/humidity, 6/6/3) is the natural spline function for time, temperature, and humidity with 6/6/3 df.

Relative risks and their confidence intervals (CIs) were estimated to quantify the influence of each air pollutant on pediatric outpatients. All results are presented as relative risk (RR) and 95% CI of daily pediatric respiratory outpatients, which were associated with every unit increase in ambient air pollutant concentration.

Data were stratified according to seasonal patterns, namely, the cool season (November to March), the hot season (June to August), and the transition season (April, May, September, and October). In addition, we built two-pollutant models to evaluate the stability of PM_2.5_ effects after adjustment for co-pollutants. Lag analysis was conducted to observe the aspect of time in revealing some delayed effects of air pollutants on pediatric respiratory outpatients. The following lag structures were used: single-day lag (0–5) and multi-day lag (01,03,05). Considering the lagged and usually non-linear relationship, we applied a distributed lag non-linear model (DLNM) to explore the influence of PM_2.5_ on pediatric respiratory outpatients (21).

All statistical tests were performed using R software (version 4.0.4) with “mgcv” (https://github.com/cran/mgcv) and “DLNM” (https://github.com/gasparrini/dlnm) packages. Statistical significance was set at *P* < 0.05. The results are presented as the percentage change of pediatric respiratory outpatient visits per μg/m^3^ increase in PM_2.5_/day.

## Results

This study extracted data from 142,825 pediatric patients from the respiratory department between January 1, 2014, to December 31, 2020. The daily average temperature and humidity were 17.58°C and 80.46%, respectively. The mean daily air pollutant concentration of CO was 0.64 mg/m^3^, the mean concentrations of other pollutants were 6.8 μg/m^3^ for SO_2_, 19.55 μg/m^3^ for NO_2_, 94.55 μg/m^3^ for O_3_, 24.89 μg/m^3^ for PM_2.5_, and 43.32 μg/m^3^ for PM_10_. The average concentrations of air pollutants and weather conditions per year were showed in Table 1. Children were affected most by the J00–J06 respiratory disease category. Significant differences were observed among disease categories between different years (*P* < 0.001).

**Table 1:** Descriptive statistics for daily outpatient visits, concentrations of air pollutants, and weather conditions.

	**2014**	**2015**	**2016**	**2017**	**2018**	**2019**	**2020**	***P* value**
	**Mean (SD)**	**Mean (SD)**	**Mean (SD)**	**Mean (SD)**	**Mean (SD)**	**Mean (SD)**	**Mean (SD)**	
Pressure (Pa)	1,012.05 (8.36)	1,012.18 (8.74)	1,011.98 (8.62)	1,012.57 (8.17)	1,011.78 (8.68)	1,011.84 (8.70)	1,012.45 (8.46)	0.847
Temperature (°C)	17.04 (7.59)	17.17 (7.40)	17.85 (8.13)	17.83 (8.11)	17.69 (8.11)	17.51 (7.36)	18.02 (7.38)	0.552
Humidity	80.14 (11.66)	80.77 (11.40)	82.30 (11.59)	79.01 (12.04)	81.92 (11.81)	81.34 (11.62)	77.76 (11.79)	<0.001
SO_2_ (μg/m^3^)	5.83 (4.56)	6.39 (3.74)	8.98 (2.88)	10.21 (2.86)	6.73 (3.28)	4.65 (1.52)	4.87 (1.70)	<0.001
NO_2_ (μg/m^3^)	22.19 (12.79)	23.42 (13.61)	19.93 (10.85)	18.03 (10.48)	17.55 (10.78)	18.28 (9.55)	17.51 (9.32)	<0.001
CO (mg/m^3^)	0.66 (0.24)	0.59 (0.25)	0.66 (0.21)	0.70 (0.20)	0.73 (0.23)	0.57 (0.15)	0.58 (0.18)	<0.001
O_3_ (μg/m^3^)	89.90 (32.09)	93.90 (32.41)	95.59 (35.15)	101.91 (34.55)	86.41 (32.77)	96.28 (31.31)	97.91 (32.35)	<0.001
PM_10_ (μg/m^3^)	57.01 (35.91)	49.43 (31.82)	44.35 (25.49)	46.03 (26.83)	39.58 (23.42)	37.12 (24.60)	29.83 (17.37)	<0.001
PM_2.5_ (μg/m^3^)	31.18 (20.67)	31.28 (22.95)	26.76 (17.62)	26.04 (16.80)	23.31 (16.61)	19.82 (14.54)	15.98 (10.97)	<0.001
J00–J06	23.73 (14.83)	29.15 (9.84)	30.51 (9.94)	29.48 (14.09)	30.37 (14.17)	34.89 (12.58)	18.44 (17.04)	<0.001
J09–J18	3.31 (3.06)	1.90 (1.90)	2.68 (2.47)	1.60 (1.81)	2.06 (2.64)	4.57 (3.41)	2.14 (4.15)	<0.001
J20–J22	5.99 (5.64)	9.68 (6.25)	11.14 (7.74)	13.84 (8.58)	18.86 (8.34)	24.21 (10.36)	8.81 (13.06)	<0.001
J30–J39	1.39 (3.07)	0.13 (0.36)	0.04 (0.19)	0.04 (0.19)	0.52 (1.57)	1.45 (3.58)	1.17 (2.58)	<0.001
J40–J47	13.30 (8.26)	10.65 (6.76)	10.32 (6.71)	7.21 (5.52)	6.98 (6.17)	5.67 (4.92)	7.31 (11.95)	<0.001
J60–J70	0.00 (0.00)	0.00 (0.00)	0.00 (0.00)	0.00 (0.00)	0.00 (0.00)	0.00 (0.00)	0.04 (0.19)	<0.001
J80–J84	0.02 (0.14)	0.00 (0.00)	0.00 (0.00)	0.00 (0.00)	0.00 (0.00)	0.00 (0.00)	0.31 (0.96)	<0.001
J85–J86	0.01 (0.09)	0.00 (0.00)	0.00 (0.00)	0.00 (0.00)	0.00 (0.00)	0.00 (0.00)	0.02 (0.17)	<0.001
J90–J94	0.09 (0.34)	0.00 (0.00)	0.00 (0.05)	0.00 (0.00)	0.00 (0.00)	0.00 (0.05)	0.10 (0.36)	<0.001
J95–J99	0.62 (1.72)	0.00 (0.00)	0.00 (0.05)	0.00 (0.00)	0.02 (0.13)	0.00 (0.05)	0.64 (1.88)	<0.001
R04	0.12 (0.39)	0.10 (0.33)	0.10 (0.32)	0.10 (0.35)	0.12 (0.34)	0.10 (0.33)	0.22 (0.53)	<0.001
R05	1.95 (4.72)	0.51 (1.44)	0.28 (0.62)	0.51 (0.83)	1.10 (1.34)	2.26 (2.49)	2.89 (4.88)	<0.001
R06	0.37 (0.67)	0.34 (0.71)	0.20 (0.49)	0.00 (0.05)	0.01 (0.12)	0.01 (0.10)	0.14 (0.55)	<0.001
R07	2.45 (5.69)	0.07 (0.29)	0.10 (0.32)	0.07 (0.26)	0.13 (0.36)	0.16 (0.41)	1.29 (2.81)	<0.001
R09.0–R09.3	0.02 (0.14)	0.00 (0.00)	0.00 (0.05)	0.00 (0.00)	0.00 (0.00)	0.00 (0.00)	0.07 (0.32)	<0.001
Respiratory	53.35 (24.79)	52.53 (16.07)	55.38 (18.97)	52.84 (22.45)	60.16 (21.95)	73.32 (22.47)	43.58 (40.88)	<0.001

*Coding from the International Classification of Diseases, 10th edition: J00–J99, respiratory diseases; J00–J06, acute upper respiratory tract infection; J09–J18, influenza and pneumonia; J20–J22, other acute lower respiratory infections; J30–J39, other diseases of the upper respiratory tract; J40–J47, chronic lower respiratory disease; J60–J70, pulmonary diseases caused by external substances; J80–J84, other respiratory diseases mainly affecting stroma; J85–J86, suppurative and necrotic condition of lower respiratory tract; J90–J94, other diseases of the pleura; J95–J99, other diseases of the respiratory system; R04, respiratory tract bleeding; R05, cough; R06, abnormal breathing; R07, sore throat and chest pain; R09, other symptoms and signs involving the circulatory and respiratory systems; R09.0, asphyxia; R09.1, pleurisy; R09.2, breathing stops; R09.3, abnormal sputum*.

**Figure 1:**
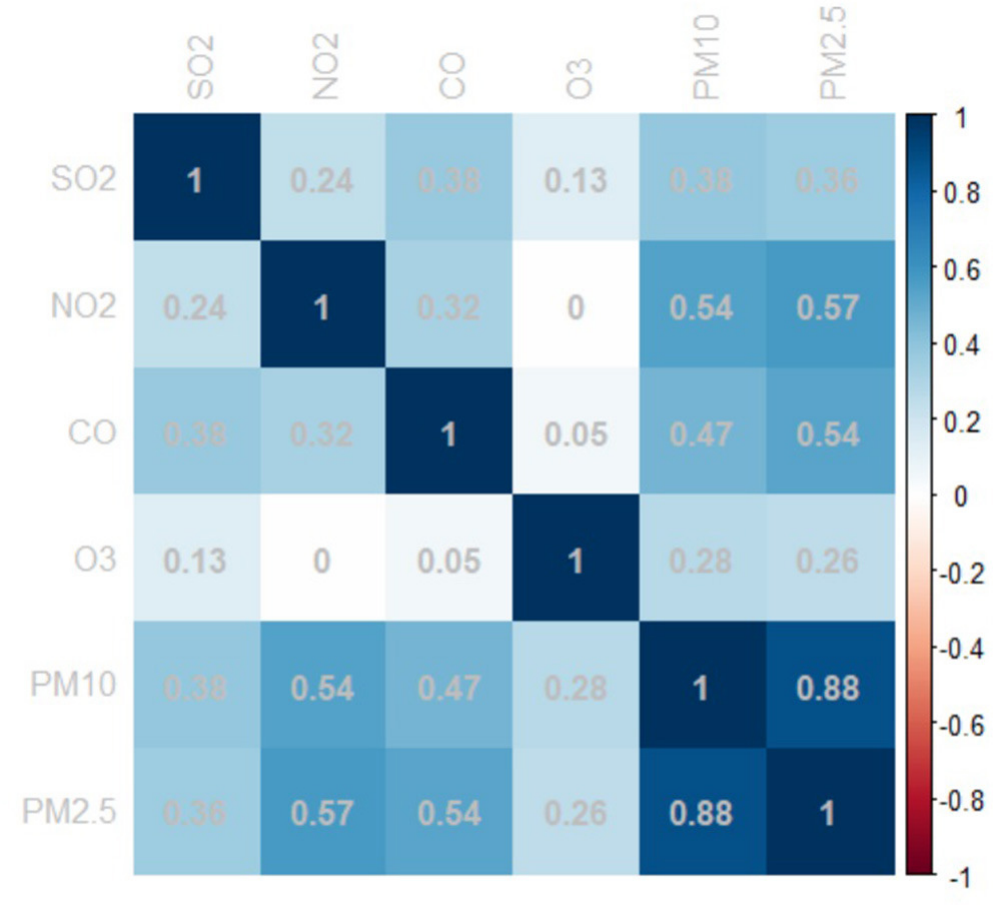
Heatmap of spearman correlation coefficients between daily ambient air pollutants.

Figure 1 shows the correlation between ambient air pollutants. A strong correlation was observed between PM_2.5_ and PM_10_ with a Pearson's correlation coefficient of 0.885. Additionally, PM_2.5_ was correlated with CO, and SO_2_ was positively correlated with PM_2.5_, PM10, NO_2_, and CO, which suggest collinearity of these variables.

The lag model was used to explore the relationship between air pollution and outpatient volume since they exhibited a hysteresis effect. Table 2 shows the correlations between air pollutants and daily pediatric outpatients on single-lag (lag0–lag5) and multi-lag days (lag01–lag03). PM_2.5_ was significantly associated with daily pediatric outpatients in the single and multi-pollutant models, either from single-lag or multi-lag models. The correlation coefficient fluctuated between 0.999 and 1.007.

**Table 2:** Relative risk (RR) for significant associations between air pollutions and daily respiratory outpatient visits due to lag effects.

	**PM_2.5_**	**PM_2.5_+SO_2_**	**PM_2.5_+CO**	**PM_2.5_+NO_2_**	**PM_2.5_+O_3_**
	**RR (95%CI)**	**RR (95%CI)**	**RR (95%CI)**	**RR (95%CI)**	**RR (95%CI)**
lag0	1.003 (1.003–1.003)	1.002 (1.002–1.002)	1.003 (1.003–1.003)	1.000 (1.000–1.000)	1.004 (1.004–1.004)
lag1	1.003 (1.002–1.003)	1.002 (1.001–1.002)	1.003 (1.003–1.003)	0.999 (0.999–1.000)	1.003 (1.003–1.004)
lag2	1.002 (1.002–1.003)	1.002 (1.001–1.002)	1.003 (1.003–1.003)	1.000 (0.999–1.000)	1.003 (1.003–1.004)
lag3	1.002 (1.002–1.003)	1.002 (1.001–1.002)	1.003 (1.002–1.003)	0.999 (0.999–1.000)	1.003 (1.003–1.004)
lag4	1.002 (1.002–1.003)	1.002 (1.001–1.002)	1.002 (1.002–1.003)	0.999 (0.999–1.000)	1.003 (1.003–1.004)
lag5	1.002 (1.002–1.002)	1.001 (1.001–1.001)	1.002 (1.002–1.002)	0.999 (0.998–0.999)	1.003 (1.003–1.003)
lag01	1.004 (1.003–1.004)	1.003 (1.002–1.003)	1.004 (1.004–1.004)	1.000 (0.999–1.000)	1.005 (1.005–1.005)
lag03	1.005 (1.005–1.006)	1.004 (1.003–1.004)	1.005 (1.005–1.006)	0.999 (0.999–1.000)	1.007 (1.007–1.008)
lag05	1.003 (1.003–1.003)	1.002 (1.002–1.002)	1.003 (1.003–1.003)	1.000 (1.000–1.000)	1.004 (1.004–1.004)

**Figure 2:**
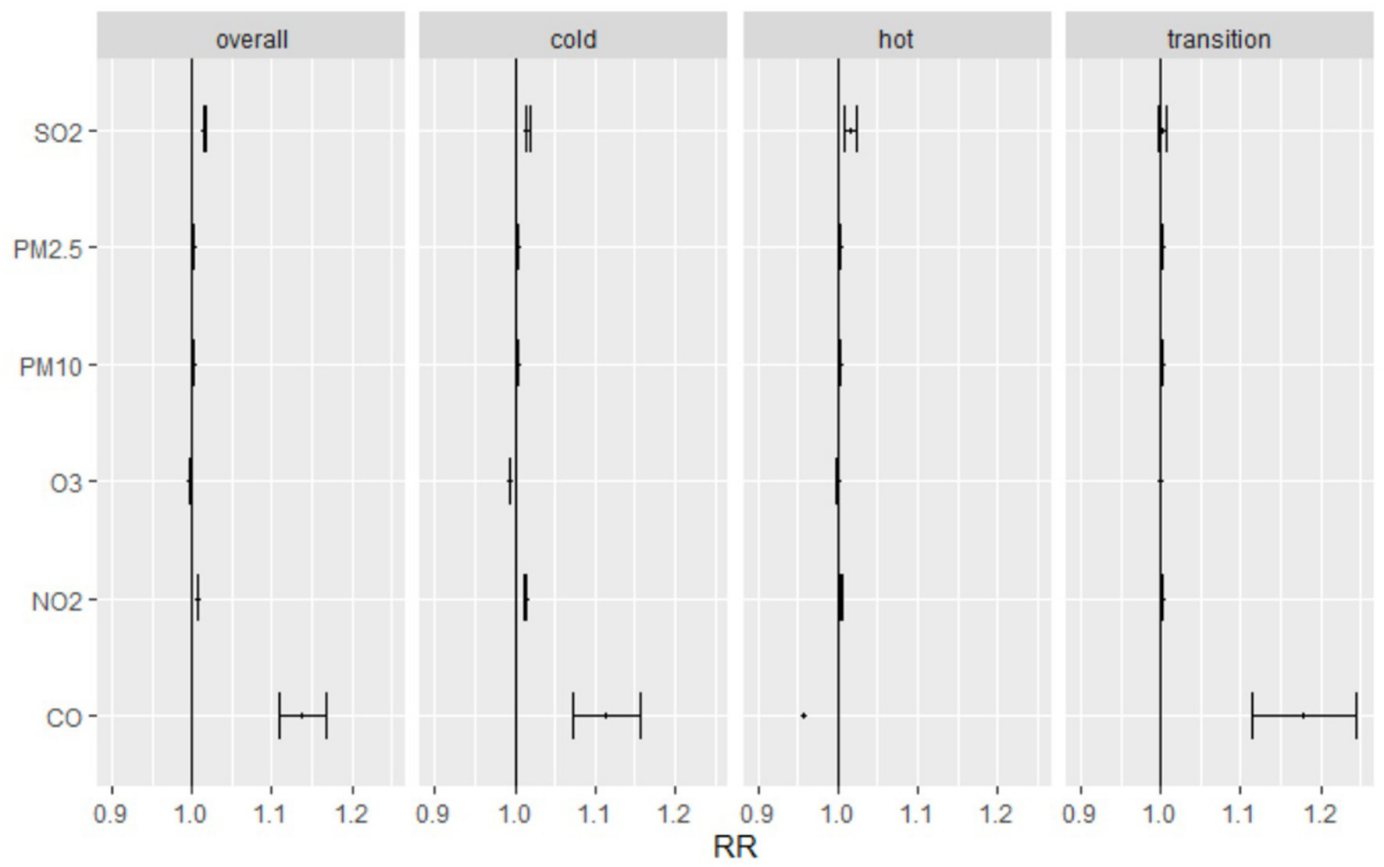
Relative risk and 95% confidence intervals for daily pediatric respiratory outpatients with per unit increase of PM_2.5_, PM_10_, SO_2_, O_3_, NO_2_ and CO in hot, cold, transition seasons. Cold Season: November to March; Hot Season: June to August; Transition Season: April, May, September, and October.

The temperature in different seasons affected the number of pediatric outpatient visits. The seasons and effects estimates of different seasons were stratified into three categories according to the mean and 95% CI of daily visits per unit increase in PM_2.5_, PM_10_, SO_2_, O_3_, and NO_2_ (Figure 2). In all seasons, daily pediatric outpatients were significantly correlated with increased PM_2.5_, PM_10_, SO_2_, O_3_, and NO_2_. In the cold environment, CO was similar to the number of pediatric respiratory outpatients and the other pollutants; on the other hand, CO was negatively correlated with pediatric respiratory outpatients during the hot season. In transition season, CO was highly positively correlated with pediatric respiratory outpatients while other air pollutants effects were unconspicuous.

**Figure 3:**
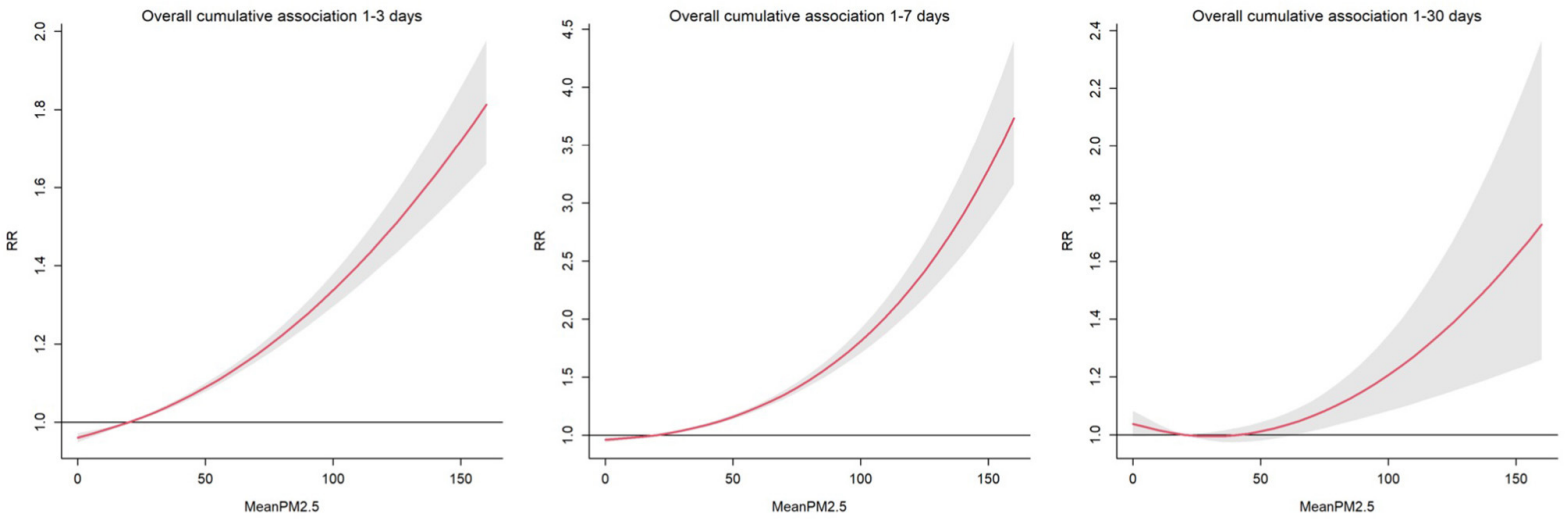
Relative risk and 95% confidence intervals for daily pediatric respiratory outpatients with per unit increase of PM_2.5_ at different cumulation lag days.

**Figure 4:**
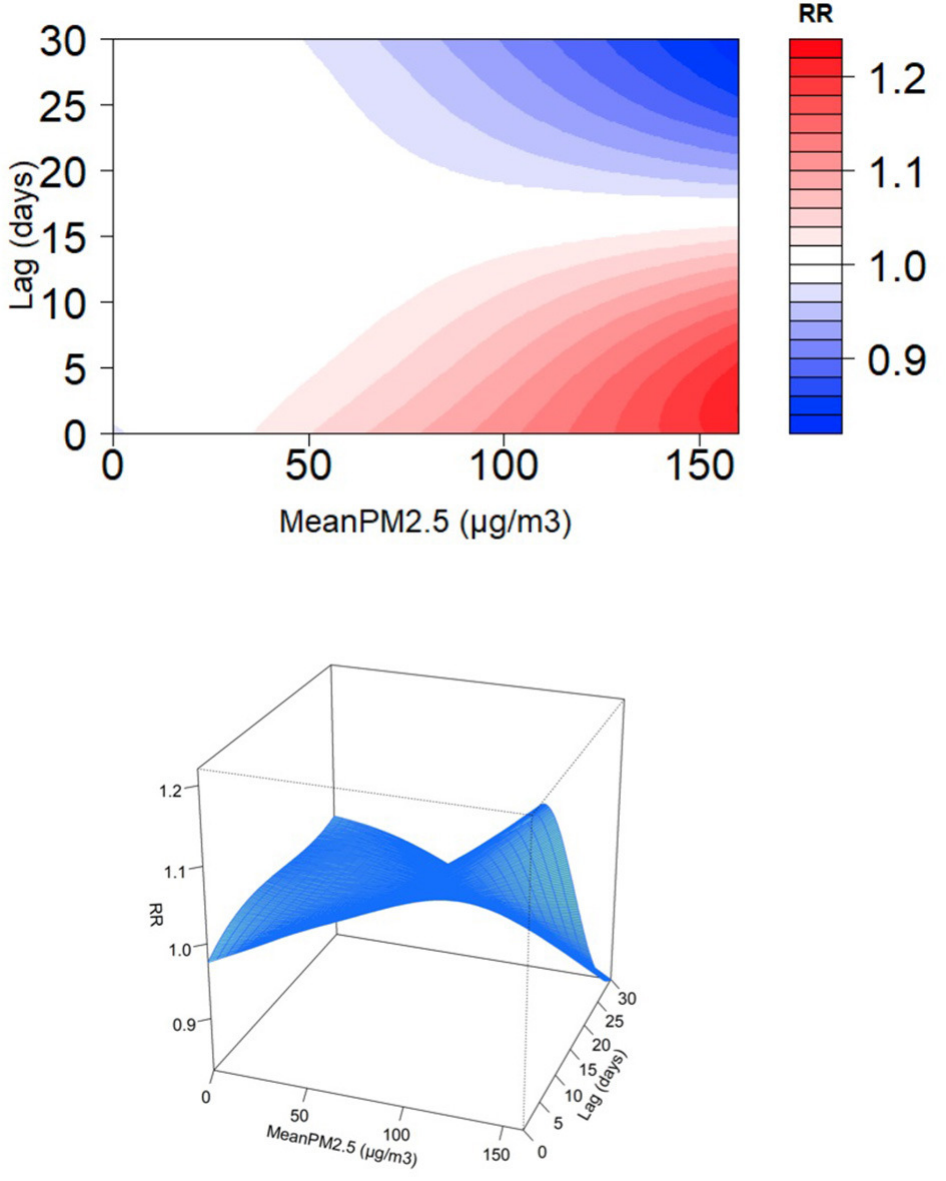
Relative risk for daily pediatric respiratory outpatients with per unit increase of PM_2.5_ at different cumulation lag days.

We explored the daily change in the RR of pediatric respiratory outpatients for an increase in the average PM_2.5_ by one microgram/m^3^ for 3, 7, and 30 days. A direct relationship was observed between the RR of pediatric respiratory outcomes and PM_2.5_ average accumulative amount and rate (Figure 3). For all cumulative lag days, PM_2.5_ greater than 25 μg/m^3^ was a consistent risk factor (22); particularly, a direct relationship was observed between PM_2.5_ concentration and respiratory risk in children. Figure 4 shows the RR increase of PM_2.5_ per unit in pediatric respiratory outpatients on different cumulative lag days. In lag days <15 days, a direct relationship was observed between PM_2.5_ concentration and RR. When the lag days exceeded 15 days, the lag effect of PM_2.5_ began to weaken.

## Discussion

### Clinical Implications

This is one of the few studies exploring the relationship between the number of pediatric respiratory outpatient visits and air pollution in developing country. Previous studies, most in developed country, have discussed the impact of air pollutants, such as NO_2_ and PM_2.5_, on the development of asthma in children (23–25). Other studies have found that changes in air quality affect the development of respiratory diseases in children, with an incidence rate ratio of ~0.8 for NO_2_ and PM_2.5_ (26), consistent with other studies in north China (13, 14, 27, 28). However, the ocean currents, climate, and economic development in south coastal cities have led to variations in the type and components of pollutants in different places (29). Another US study found a difference in PM_2.5_ levels between indoors and outdoors (29); however, we found that PM_2.5_ levels were five times elevated. Therefore, three important points are emphasized in this study. First, this study fills the gap of studies focus on the relationship between air pollution and children respiratory diseases in southern Chinese cities. Second, this study emphasized the impact of air pollutants in coastal cities, represented by Zhoushan, and the children with respiratory diseases. Third, the outpatient data of children with respiratory diseases were used. This was a comprehensive overview of all possible variations in childhood respiratory diseases. We used ICD codes to classify diseases to avoid missing relevant diagnoses.

Long-term and short-term exposure to fine particulate matter, such as PM_2.5_ and PM_10_, may indirectly affect the central nervous system and gastrointestinal tract (30). The small size of the particles allows easy penetration of the body's immune barriers, causing acute and chronic diseases and even premature death (31–33). A synergy exists between these major air pollutants, which travel from the nasal cavity to the trachea or the lungs, where they interact with each other. The mucosal layer, epithelial tissue, or respiratory tract cells coordinate with immune cells such as dendritic cells, macrophages, and monocytes to protect the body from pollutants. However, air pollutants can interrupt the body's homeostasis and release cytokines, which can damage respiratory health (34, 35). The air pollutant-induced excessive release of cellular inflammatory factors and immune cells may cause tissue damage and lung inflammation. Additionally, the immune system may be targeted, leading to immune dysfunction due to damage to immune cells such as natural killer cells (36–38). In addition, organic compounds in air pollutants can release electrons to form superoxides, damaging the body's antioxidants and causing inflammation (39, 40). Other studies have shown that prolonged exposure to low doses of PM_2.5_ causes mast cell overexpression, which leads to increased inflammatory cytokine and histamine production (41, 42). Overall, the number of outpatient visits was the most sensitive indicator of impaired respiratory function. In Yangcheng, China, a time-series analysis found a 1.69% increase in the number of outpatient visits for respiratory diseases caused for every 10 μg/m^3^ increase in PM_2.5−10_ (43), which was consistent with that of eastern and western cities in China (44, 45). These findings prove the relationship between air pollutants and the number of outpatient visits.

Additionally, air pollution and temperature exhibit a synergistic effect. Studies have found that air pollutants in drought conditions can exacerbate chronic respiratory diseases and cause acute reactions (46, 47). Our study found differences in pollutant concentrations at different temperatures. Notably, carbon monoxide concentration was highest in the cold season but lowest in the hot season. This may be attributed to the status of Zhoushan city as a coastal city, where residents burn coal for outdoor heating in the cold season. There was no relationship observed between the mean annual air temperature and the annual trend of pollutant concentration, suggesting the presence of many unknown confounding factors between temperature change and pollutant concentration.

In this study, we found that short-term exposure (within 1 month) to PM_2.5_ was significantly associated with increased pediatric respiratory outpatient visits, especially in the lag mode of 1–7 days. In addition, the lag patterns of 1–3 and 1–30 days showed significantly rising levels. Jiang et al. found a significant association between coarse particulate matter and a 5-day lag in respiratory and cardiovascular outpatient visits (43), Other studies have shown similar results, such as PM_2.5_ being associated with a a-day lag in asthma visits and PM_2.5−10_ being associated with 1-day lag mortality (48, 49). We observed that an increase in PM_2.5_ concentration by 10 μm/m^3^ resulted in ~4 and 10% in pediatric respiratory clinic visits for a lag of 1–3 days and 1–7 days, respectively. This is much higher than the 1% to 3% observed in previous studies and may be attributed to the growth and development period of the children's respiratory system. During this period, children's lungs are more vulnerable to air pollutants compared to adults. The lag time we observed was between 1 and 7 days, which is similar to previous studies. Additionally, the lag model of pollutant synergy revealed that the combination of PM_2.5_ and O_3_ reached a maximum value of 0.7% at lag03, which reached 1.6% in the Yancheng study. This may be attributed to the relatively low level of O_3_ and the weak coordination effect of PM_2.5_ caused by Zhoushan city being a coastal city with a lower industrial level and vehicle number than Yancheng.

Lower concentrations of PM_2.5_ caused a gentle decrease in slope (Figure 3); in contrast, an increasing slope was observed with increased PM_2.5_ concentration. This finding may be due to the small size of PM_2.5_; furthermore, it is mainly deposited in the lower respiratory tract, and a high concentration is required to reach the lungs. However, further randomized clinical trials are needed to validate these results.

The reason we choosed DLNM, which is a non-parametric model, based on following two points. Firstly, we discovered that the distribution of scatter plot is a non-linear wavy curve; Secondly, the DLNM takes account of both lag effects and the exposure-reaction non-linear relationship, and initially applied in epidemiological studies in 2006 (50). Multiple studies have suggested that the effects of air pollution and meteorological factors on outcomes have lag effects, while traditional linear models, which only study effects on certain time points without considering lag effects, are likely to produce high linearity, biasing the results (51, 52). Therefore, DLNM is a better choice to study the lag effects of exposure, especially in the analysis of air pollution. This study also use GAM with smooth spline function to fit variables in order to reduce the model risk caused by linear settings. Which means predictor variable in the model will be divided into multiple parts, and then fits each part separately through a polynomial function.

### Limitations

This study has several limitations. First, exposure error bias greatly affects time series analysis, limiting the extrapolation of this study's inferences. Second, we used outpatient data; therefore, misclassification bias was inevitable. Third, the cities included were located in a specific region, which may affect the generalizability of the results. Future studies using larger sample sizes over a wider range of regions are needed to validate the findings of this study.

## Conclusions

This study provides evidence regarding the effect of air pollutants on children's respiratory function in coastal cities. Particularly, an increase in PM_2.5_ increased the number of outpatient children in respiratory clinics. Furthermore, different lag effects were correlated with different exposure levels. These findings can improve Zhoushan's medical service and health risk assessment policies, providing implications for other similar places or countries.

## Data Availability Statement

The original contributions presented in the study are included in the article/supplementary material, further inquiries can be directed to the corresponding author.

## Ethics Statement

The studies involving human participants were reviewed and approved by Institutional Review Board of the Shanghai International Medical Center (Approval Number: S20210513). Written informed consent to participate in this study was provided by the participants' legal guardian/next of kin.

## Author Contributions

W-YL, J-PY, LS, and T-HT conducted the study and drafted the manuscript. W-YL and J-PY participated in the design of the study and performed data synthesis. LS and T-HT conceived the study and participated in its design and coordination. All authors read and approved the final manuscript.

## Conflict of Interest

The authors declare that the research was conducted in the absence of any commercial or financial relationships that could be construed as a potential conflict of interest.

## Publisher's Note

All claims expressed in this article are solely those of the authors and do not necessarily represent those of their affiliated organizations, or those of the publisher, the editors and the reviewers. Any product that may be evaluated in this article, or claim that may be made by its manufacturer, is not guaranteed or endorsed by the publisher.
